# Meta-Inflammation and Metabolic Reprogramming of Macrophages in Diabetes and Obesity: The Importance of Metabolites

**DOI:** 10.3389/fimmu.2021.746151

**Published:** 2021-11-05

**Authors:** Sara Russo, Marcel Kwiatkowski, Natalia Govorukhina, Rainer Bischoff, Barbro N. Melgert

**Affiliations:** ^1^ Department of Analytical Biochemistry, University of Groningen, Groningen, Netherlands; ^2^ Department of Biochemistry and Center for Molecular Biosciences Innsbruck, University of Innsbruck, Innsbruck, Austria; ^3^ Department of Molecular Pharmacology, University of Groningen, Groningen, Netherlands; ^4^ Groningen Research Institute for Asthma and COPD (GRIAC), University Medical Center Groningen, Groningen, Netherlands

**Keywords:** M1, classically activated macrophage, M2, DMTII, metabolite analysis, MS, metabolic syndrome, alternatively activated macrophage

## Abstract

Diabetes mellitus type II and obesity are two important causes of death in modern society. They are characterized by low-grade chronic inflammation and metabolic dysfunction (meta-inflammation), which is observed in all tissues involved in energy homeostasis. A substantial body of evidence has established an important role for macrophages in these tissues during the development of diabetes mellitus type II and obesity. Macrophages can activate into specialized subsets by cues from their microenvironment to handle a variety of tasks. Many different subsets have been described and in diabetes/obesity literature two main classifications are widely used that are also defined by differential metabolic reprogramming taking place to fuel their main functions. Classically activated, pro-inflammatory macrophages (often referred to as M1) favor glycolysis, produce lactate instead of metabolizing pyruvate to acetyl-CoA, and have a tricarboxylic acid cycle that is interrupted at two points. Alternatively activated macrophages (often referred to as M2) mainly use beta-oxidation of fatty acids and oxidative phosphorylation to create energy-rich molecules such as ATP and are involved in tissue repair and downregulation of inflammation. Since diabetes type II and obesity are characterized by metabolic alterations at the organism level, these alterations may also induce changes in macrophage metabolism resulting in unique macrophage activation patterns in diabetes and obesity. This review describes the interactions between metabolic reprogramming of macrophages and conditions of metabolic dysfunction like diabetes and obesity. We also focus on different possibilities of measuring a range of metabolites intra-and extracellularly in a precise and comprehensive manner to better identify the subsets of polarized macrophages that are unique to diabetes and obesity. Advantages and disadvantages of the currently most widely used metabolite analysis approaches are highlighted. We further describe how their combined use may serve to provide a comprehensive overview of the metabolic changes that take place intracellularly during macrophage activation in conditions like diabetes and obesity.

## Introduction

Diabetes mellitus type II (DMTII) is one of the main causes of death in modern society according to the World Health Organization (1). It correlates with long-term complications that include nephropathy, peripheral neuropathy, and cardiovascular disease. The International Diabetes Federation has estimated that globally the diagnosis of DMTII has been made in 415 million people and anticipates growth to up to 642 million by the year 2040 (2).

Several factors can contribute to a higher risk of developing DMTII, but it has been proven that overweight or obesity are the most important ones (3). DMTII is often linked to obesity and both are associated with metabolic syndrome, which encompasses conditions such as high blood pressure, excess body fat around the waist, high blood sugar, high serum cholesterol or triglyceride levels, and low high-density lipoprotein (HDL) cholesterol. Metabolic syndrome is characterized by low-grade chronic inflammation (meta-inflammation) (4) in all tissues involved in energy homeostasis, including adipose tissue, pancreatic islets, and liver (5). Studies have shown that the metabolic consequences of adipose tissue dysfunction increase mortality in patients with DMTII, emphasizing the importance of meta-inflammation in the context of DMTII (6)

Macrophages are part of the innate immune system and are present in all tissues of our body, including adipose tissues (7). They play a crucial role in the first line of defense against microorganisms and other external or internal threats to homeostasis by initiating essential inflammatory responses (8). These inflammatory responses are facilitated by changes in macrophage cellular metabolism, with a focus on glycolysis that is induced in cells producing inflammatory mediators. The inflammatory response is counter-balanced by stimulation of tissue repair and anti-inflammatory mechanisms once the threat has been overcome. At the same time, the cellular metabolism changes from glycolysis to oxidative phosphorylation to aid in tissue repair. Continuous exposure to pro-inflammatory stimuli, however, can shift the balance of inflammation and repair in favor of chronic inflammation and tissue damage. Excessive activation of macrophage inflammatory responses is seen in many diseases characterized by the continuous presence of pro-inflammatory stimuli, including DMTII, and explains in part the meta-inflammation found in this condition.

Many studies have described how macrophages become activated by inflammatory stimuli (9, 10) and there is increasing consensus that a particular macrophage activation state is associated with DMTII. Characterization of the different macrophage activation states is complicated, but in recent years has been aided by the development and use of novel techniques like multiparametric flow cytometry, single-cell RNA sequencing, and real-time extracellular flux analysis. Especially the latter has the potential to improve our understanding of how macrophages can switch between different types of responses. In DMTII and obesity, the changes in macrophage cellular metabolism coincide with profound changes in metabolism on a tissue and organism level, that probably interact and give rise to a specific DMTII-associated macrophage activation state (11). This review aims to summarize what is currently known about macrophage activation in DMTII-related meta- inflammation, how changes in intracellular metabolism are influenced by the changed presence in extracellular nutrients and metabolites, and how fluctuations in key metabolic intermediates could also play a role in cellular processes like gene expression. This overview emphasizes that profiling metabolites can help to characterize macrophages and their responses and to understand how changes in their intracellular metabolites affect DMTII progression. Therefore, we finish with a comparison between different approaches to metabolite analysis to provide an overview of the currently available methods and their pros and cons, highlighting metabolomics studies that have made use of these methods and have been central to characterizing macrophages.

## Insulin Resistance and Inflammation

**Figure 1:**
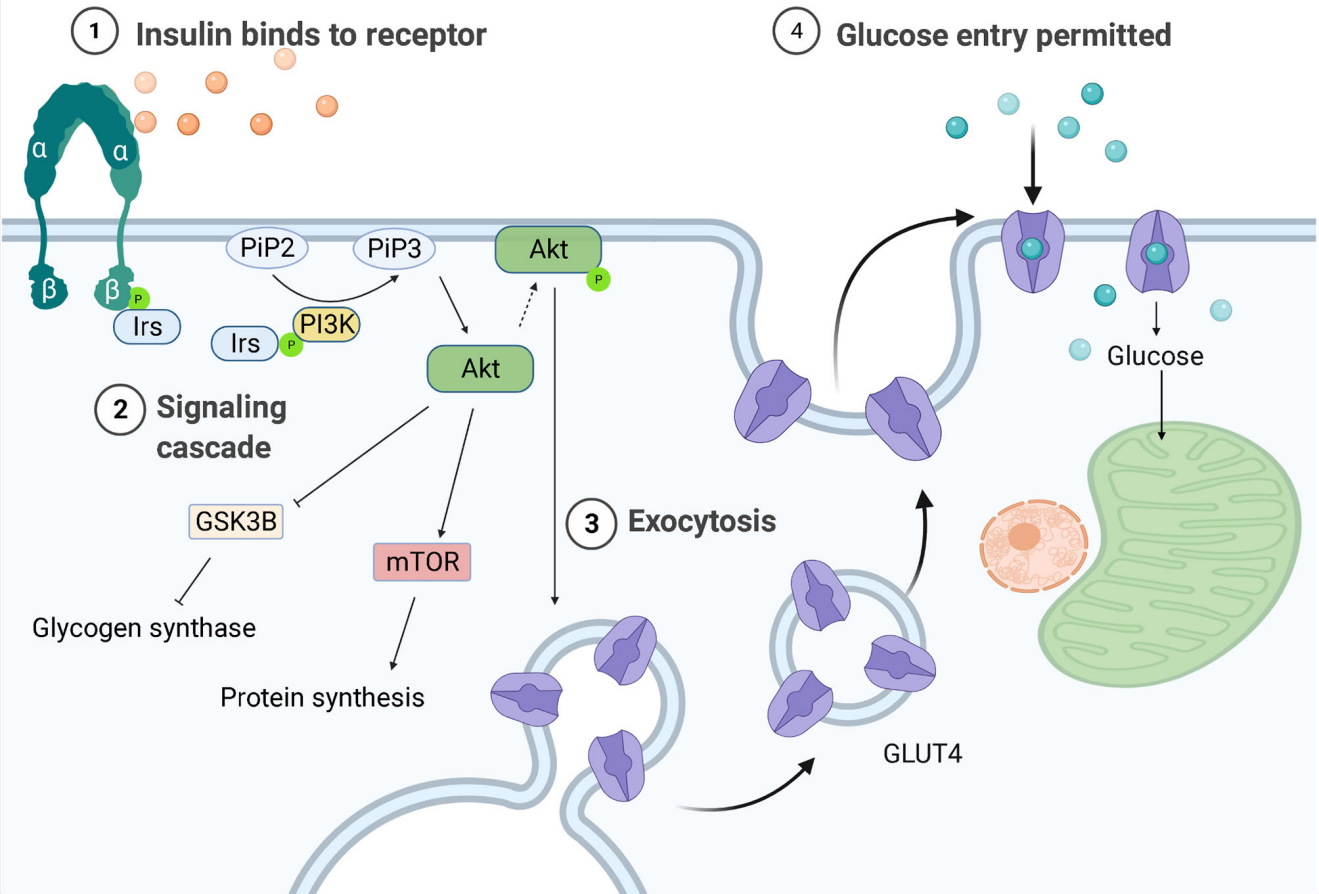
Regulation of glucose entrance through insulin signaling. Insulin receptors are tyrosine kinases consisting of two extracellular α-subunits and two transcellular β-subunits. In healthy individuals, insulin will bind the α subunit of the insulin receptor, causing a conformational change that leads to phosphorylation of tyrosine residues in its β subunit. The proteins insulin receptor substrates 1 or 2 (Irs-1/-2) will then bind to the tyrosine-phosphorylated region of insulin receptors and be themselves phosphorylated. Phosphoinositide-3-kinase (PI3K) will bind to the phosphorylated IRS-1 or -2 and be activated, producing 3-phosphorylated polyphosphoinositides (PiP3) from phosphatidylinositol 4,5-bisphosphate (PiP2). PiP3 will recruit the serine/threonine kinase Akt (also known as protein kinase B) from the cytosol to the plasma membrane, where it will be phosphorylated and activated, leading to glycogen synthase kinase-3β (GSK3B) inhibition and therefore to higher glycogen synthesis. AKT is also responsible for the translocation of the glucose transporter (GLUT4) to the plasma membrane, allowing glucose entry.

One of the key characteristics of DMTII is the altered insulin response. In healthy individuals, with a body mass index (BMI) in the normal range, pancreatic β cells produce insulin in response to circulating glucose levels. This will bind and activate insulin receptors on the cell membrane of different cell types, including macrophages, to lower blood glucose levels by enhancing its uptake by these cells. The binding of insulin to its receptor drives a cascade of events ultimately leading to uptake of glucose and further downstream effects (**Figure 1**). First glucose transporters, GLUT4 in most cell types and GLUT1 in macrophages, will either translocate from vesicles in the cytoplasm to the cell surface or their expression is upregulated, both increasing glucose influx into cells up to 10 times (12). Mammalian target of rapamycin (mTOR) will then be activated and protein synthesis will be induced. Furthermore, glycogen synthase kinase-3β (GSK3B) is inhibited allowing the activation of glycogen synthesis. When GSK3B is activated, it phosphorylates and inactivates glycogen synthase, decreasing glycogen synthesis, therefore GSK3B inhibition by Akt results in higher glycogen production. A change in gene transcription will also be initiated: expression of genes that favor either the synthesis of glycogen from glucose in the liver and muscles or of triglycerides from free fatty acids (FFA) in adipocytes will be induced and expression of genes that favor glycolysis will be transiently inhibited (13).

DMTII is caused by the development of insulin resistance, meaning the inability of cells to respond to insulin due to a transmission blockage of the insulin receptor, mainly in muscle and liver cells. Pancreatic β-cells will at first try to compensate for the higher levels of glucose by increasing insulin production. This will eventually lead to lower glucose availability in combination with lower tissue insulin sensitivity resulting in loss of β-cell function. This will result in lower insulin secretion, which will consequently lead to a higher concentration of glucose in blood (14).

Insulin resistance can be caused by many different factors, with obesity being the most important one (14). Elevated levels of circulating free fatty acids are one of the reasons for the development of insulin resistance in obese patients. These high levels of fatty acids are caused by increased basal lipolysis in adipose tissues and this elevated concentration has been proposed to serve as a stimulus for the entry and accumulation of macrophages in adipose tissue by increasing the local production and release of pro-inflammatory cytokines and chemokines (15). High concentrations of saturated free fatty acids will also induce pro-inflammatory effects through activation of Toll-like receptors (16). A consequence of this activation is the induction of the Jun N-terminal kinase and inhibitor of κB kinase (JNK/IKK-κB) pathways, which is then followed by an inflammatory cascade. Both JNK and IKK are believed to promote insulin resistance because they phosphorylate serine/threonine residues on insulin receptor substrate (IRS) proteins. By phosphorylating these residues, their phosphorylation by insulin receptors is blocked, which prevents the activation of insulin receptors by insulin. The result is inhibition of insulin-driven signal transduction and downstream effects thus inhibiting glucose entry into the cell and its accumulation in the blood.

## Macrophages and Inflammation in Obesity and DMTII

The inflammation related to obesity was first described in 1993 when Hotamisligil et al. showed that adipose tissue from obese rats expressed more tumor necrosis factor-α (TNF-α) (17) than adipose tissue from lean animals. Weisberg and colleagues further showed that TNF-α was not secreted by adipocytes but by macrophages and that the number of macrophages increased in adipose tissue during weight gain (10). Macrophages develop either from self-renewing fetal progenitors that can populate tissues before birth and maintain their numbers after birth or from circulating monocytes recruited to tissues after birth (18). Studies have shown a higher number of macrophages in white adipose tissue of obese subjects compared to people of normal BMI, going from 10% of total cells to more than 50% (19). The origin of these macrophages, either through local proliferation or monocyte recruitment, remains to be established in detail. A recent study in mice found that local proliferation of adipose tissue-resident macrophages at least contributes to macrophage accumulation during obesity too (20).

Studies have shown that during obesity, triglyceride accumulation causes stress on adipocytes due to an increase in cell size and subsequent hypoxia (21). Capillary network development cannot keep up with fat mass expansion, resulting in adipocytes that are too far away from the vasculature to be efficiently supplied with oxygen (22). This leads to higher expression of hypoxia-inducible factor, adipocyte activation, and production and subsequent release of free fatty acids and pro-inflammatory mediators such as interleukin-1β (IL-1β), IL-6, macrophage migration inhibitory factor (MIF), monocyte chemoattractant protein 1 (MCP-1, also known as CCL2), as well as reactive oxygen species (ROS). Further studies showed that ROS, together with the increased adipocytes size, will induce endoplasmic reticulum stress, leading to a pro-inflammatory and insulin-resistant phenotype in adipocytes (23). The pro-inflammatory mediators were found to induce recruitment of circulating monocytes and accumulation of adipose tissue macrophages (24).

Macrophages can respond in many different ways to stimuli from their microenvironment. In the past, this was described as macrophage activation, but since the discovery of the many different types of responses of macrophages, this is also called macrophage polarization (25). Polarized macrophages were broadly classified into two main groups, i.e. classically activated (or M1) macrophages and alternatively activated (or M2) macrophages, similar to the T helper 1/T helper 2 (Th1/Th2) dichotomy of helper T lymphocytes (26). However, it is now recognized that this view is too simplistic and that polarization states are better described as a continuous spectrum of responses (27).

**Figure 2:**
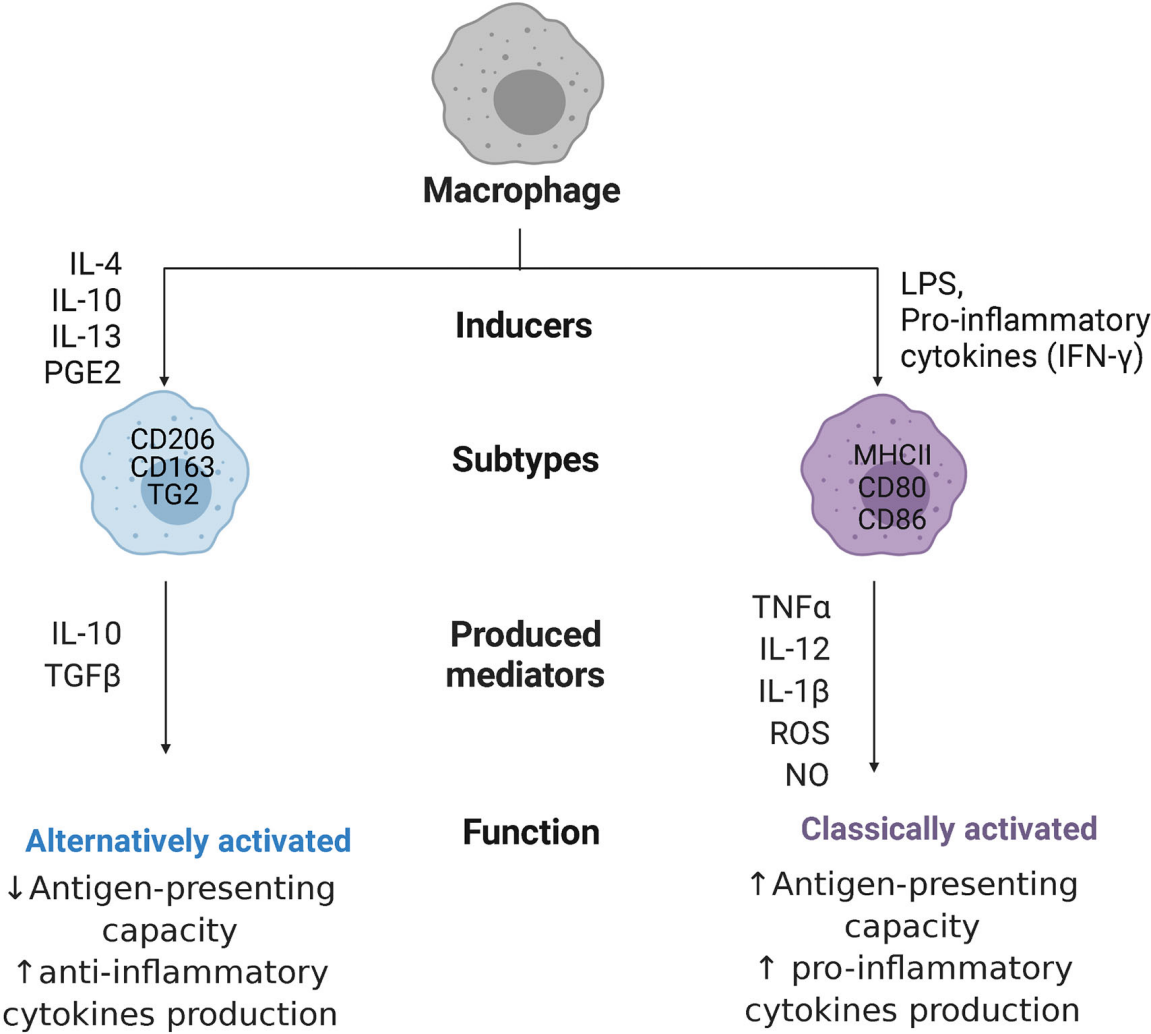
Macrophage polarization. Macrophages can polarize to classically activated macrophages, when stimulated with pro-inflammatory cytokines like interferon-ƴ (IFN-ƴ) or with bacterial products (LPS, lipopolysaccharides), or alternatively activated macrophages, when stimulated with interleukins 4,10, 13 (IL-4/10/13), or prostaglandin E2 (PGE2). Classically activated macrophages express major histocompatibility complex class II (MHC II) proteins and co-stimulatory molecules CD80 and CD86, while alternatively activated macrophages are characterized by high expression of mannose receptors CD206, high-affinity scavenger receptors CD163, and transglutaminase 2 (TG2). These cells produce, respectively, pro-inflammatory cytokines like tumor necrosis factor-α (TNF-α), IL-12, IL-1β, and IL-23 together with reactive oxygen species (ROS) and nitric oxide (NO) or anti-inflammatory cytokines like transforming growth factor β (TGFβ), and IL-10, with opposite capacity in presenting antigens.

Macrophage responses are currently mostly described by a combination of expression of extracellular and intracellular markers, including production of specific cytokines. The most widely studied and longest known activation response, i.e. classical activation, is induced by pro-inflammatory stimuli generated by infections with micro-organisms (25). These classically activated macrophages (often still called M1 macrophages) possess high antigen-presenting capacity and high potency to produce pro-inflammatory cytokines such as tumor necrosis factor-α (TNF-α), interleukin-12 (IL-12), IL-1β, and IL-23, as well as toxic mediators, such as ROS and nitric oxide (NO) (28). This type of response induces and supports Th1 responses (26). Phenotypically, classically activated macrophages express high levels of major histocompatibility complex class II (MHC II) proteins, and co-stimulatory molecules CD80 and CD86 in humans (7, 29), as shown in **Figure 2**.

When it became clear that macrophages could also become activated in ways not resembling the classical way, studies have been trying to delineate these different types of activation states. Many different types of stimuli induce slightly different alternatives to classical activation that are mostly involved in stimulating tissue repair and downregulating inflammation. These stimuli include IL-4 and IL-13, glucocorticosteroids, prostaglandin E2 (PGE2), immune complexes, transforming growth factor β (TGFβ), and IL-10 (30). These alternatively activated macrophages are associated with physiological and pathological tissue remodeling (e.g. fibrosis) and can have anti-inflammatory effects by secreting high levels of IL-10 and TGFβ. Depending on the stimulus, these macrophages (often still called M2 macrophages) are characterized by high expression of mannose receptors (CD206), high-affinity scavenger receptors (CD163), and transglutaminase 2 in humans (31) and they have poor antigen-presenting capabilities (32). These M2 macrophages have been further divided into subgroups, such as wound-healing and regulatory macrophages (33), or into M2a, M2b, M2c, and M2d subtypes (34), which rendered the nomenclature in the field more complex and has been advised against (25). The advent of new techniques like single-cell transcriptomics (35), proteomics (36), and metabolomics are helping to understand the enormous diversity in macrophages present in tissues and will hopefully lead to better classifications, although this point has not been reached yet. For the purpose of this review, the M1 and M2 nomenclature will only be used when citing studies that use these names.

Adipose tissue macrophages are resident macrophages contributing to adipose tissue homeostasis. *In vivo*, adipose tissue macrophages from healthy mice express high levels of CD206, whereas adipose tissue macrophages from obese mice express low levels of CD206 (37) and high levels of integrin CD11c (38). These CD11c-expressing macrophages are associated with insulin resistance (39) and are situated in crown-like structures, surrounding necrotic adipocytes with the goal to remove them through exophagy. This will lead to free fatty acid and lipid internalization by macrophages and foam cells formation (40). Interestingly, it has been shown that murine bone marrow-derived macrophages can upregulate the expression of CD11c when differentiated in co-culture with normal adipocytes, underpinning the importance of the microenvironment in which macrophages grow (35). In adipose tissue, different kinds of immune cells may contribute to this change in macrophage polarization, including neutrophils through the protease elastase (41), T lymphocytes through interferon-ƴ (42), natural killer cells through TNFα and MCP1 (43), and B cells through IgG antibodies (44).

As discussed above it is clear obesity changes macrophage activation status. Even though levels of pro-inflammatory cytokines produced by macrophages in obesity are higher compared to non-obese individuals, contributing to the onset of obesity related meta-inflammation, their activation status does not coincide with a classically activated status of macrophages (36). To identify which markers characterize these macrophages, monocyte-derived macrophages were cultured in media conditioned by adipose tissue of obese mice or humans to mimic the presence of the metabolic syndrome and it was shown that these macrophages accumulate lipids and have high expression of fatty acid transporters like CD36 but do not express the markers associated with classical activation (36, 45). The presence of the CD36 marker was confirmed in adipose tissue macrophages from obese subjects and was not seen in macrophages of lean individuals. This macrophage subset is defined as metabolically activated and specific markers for this type of macrophages are suggested to be macrophage scavenger receptor 1 (Msr1), ATP-binding cassette A1 (ABCA1), and the adipose differentiation-related protein (Perilipin-2, PLIN2), in addition to CD11c and CD36 (36, 45).

## Macrophage Metabolic Reprogramming

**Figure 3:**
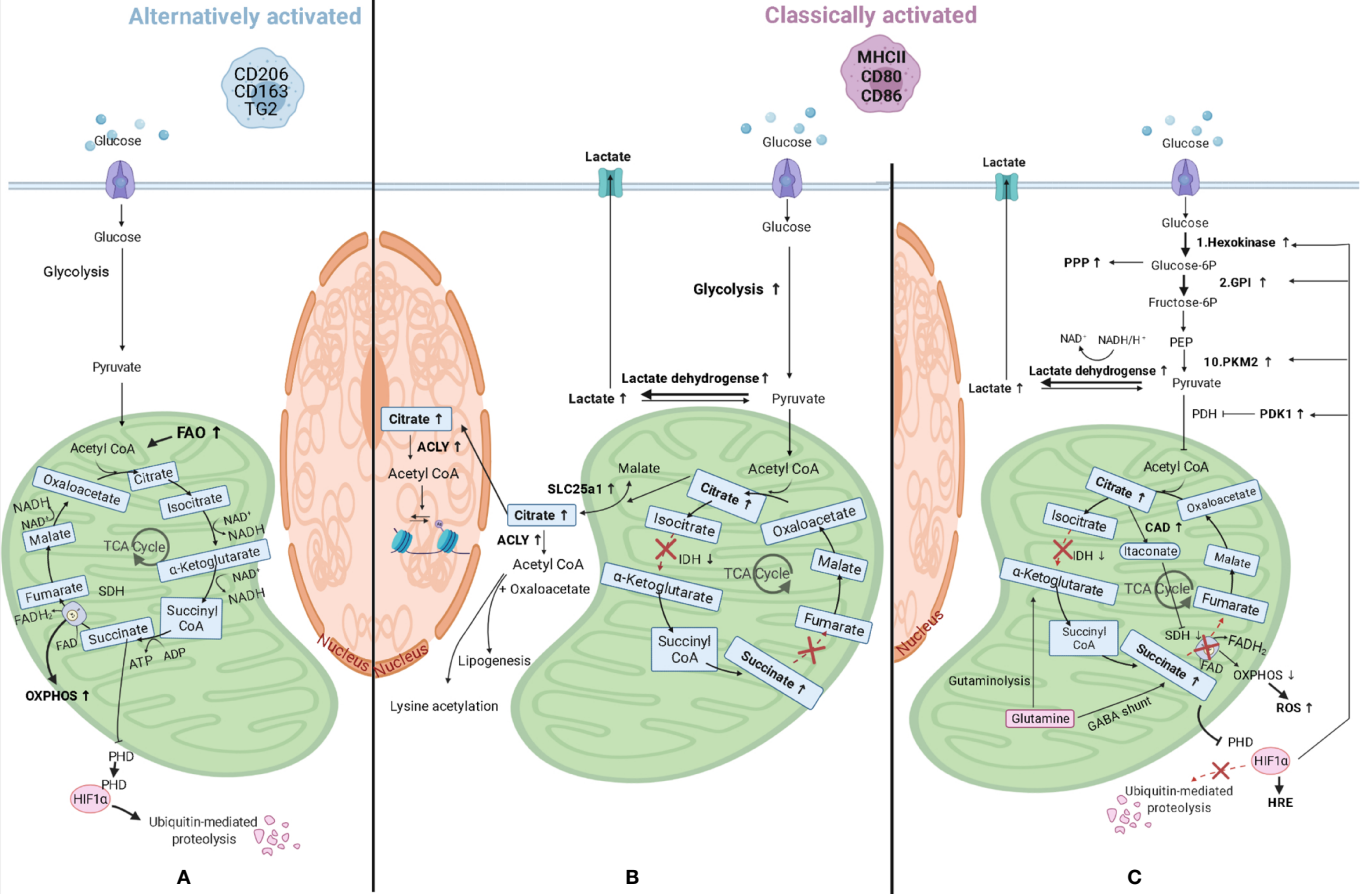
Macrophage metabolic reprograming. **(A)** Alternatively activated macrophages have an induced fatty acid oxidation (FAO) and produce pyruvate from glycolysis and this is converted in acetyl-CoA, which is then used by the tricarboxylic acid (TCA) cycle to give electrons in the form of NADH and FADH_2_ to the mitochondrial oxidative phosphorylation (OXPHOS) complexes to produce ATP. **(B)** When macrophages polarize to classically activated macrophages, metabolic reprogramming takes place and lactate is produced instead of pyruvate, and the TCA cycle is broken at two points, after citrate, and after succinate, resulting in the accumulation of these three metabolites. Citrate accumulates due to lower expression of isocitrate dehydrogenase (IDH) and can either be transported to the cytosol through solute carrier family 25 member 1 (SLC25a1), where it can be converted in acetyl-CoA by ATP citrate lyase (ACLY), or to the nucleus where the same conversion can take place. Acetyl-CoA can then be used for lysine acetylation or for lipogenesis. **(C)** Other changes in classically activated macrophages include succinate dehydrogenase (SDH) inhibition by itaconate, which is produced by upregulated cis-aconitate decarboxylase (CAD), and this results in succinate accumulation. Succinate levels can also increase as a consequence of augmented levels of glutamine anaplerosis, either through an upregulated GABA (γ-aminobutyric acid) shunt or through glutaminolysis. SDH is also part of the mitochondrial respiratory chain and its inhibition will lead to decreased mitochondrial respiration and increased ROS (reactive oxygen species) production. Succinate inhibits prolyl hydroxylase domain (PHD) proteins, resulting in less hydroxylation of hypoxia-inducible factor 1-alpha (HIF-1α), which circumvents its degradation and allows its binding to hypoxia response elements (HRE) on target genes (48). HIF-1α also promotes the switch to glycolysis by inducing glycolytic enzymes like hexokinase 2 (1^st^ reaction of glycolysis), pyruvate kinase M2 (PKM2, 10^th^ reaction), and glucose-6-phosphate isomerase (GPI, 2^nd^ reaction). The enzyme product of the latter is used in the oxidative phase of the pentose phosphate pathway (PPP), which is also upregulated in classically activated macrophages. HIF-1α also upregulates the enzymes lactate dehydrogenase and pyruvate dehydrogenase kinase 1 (PDK1) leading to higher lactate production and lower acetyl-CoA synthesis, respectively. PEP, Phosphoenolpyruvate; PDH, Pyruvate dehydrogenase.

Since macrophages are key sentinel cells in charge of detecting alterations in their microenvironment, they need to be able to respond rapidly. To do so, they also need flexible metabolic pathways and must be able to reprogram their metabolism for proper activation and function. In fact, when macrophages polarize to a different phenotype, they also modify how they process their energy substrates, such as glucose or fatty acids. One of the first differences shown in macrophage metabolism related to polarization differences was seen in amino acid metabolism, in which classically activated macrophages were found to convert arginine to NO and citrulline by inducible NO synthase (iNOS) activity, while alternatively activated macrophages convert arginine in proline and polyamines through arginase-1 (46). Following this initial observation, our knowledge has expanded and it is now known that macrophages can also reprogram the way they generate ATP for energy. Nonpolarized or alternatively activated macrophages are involved in processes that are less time-pressured and use beta-oxidation of fatty acids and mitochondrial oxidative phosphorylation (OXPHOS) to produce ATP. This is achieved by lipolysis of triglycerides (47), generating fatty acids that will be oxidized by beta-oxidation, and obtaining acetyl-CoA plus NADH and FADH_2_. The first will enter the tricarboxylic acid (TCA) cycle, while the latter are used to produce ATP by OXPHOS. In addition, these macrophages can produce pyruvate from glycolysis, convert this to acetyl-CoA, which is then used by the TCA cycle to give electrons in the form of NADH and FADH_2_ to the OXPHOS complexes (**Figure 3A**). In response to pro-inflammatory stimuli, macrophages reprogram their metabolism to create energy and biosynthetic precursors rapidly in order to fight fast-growing microbes. This phenomenon is similar to the Warburg effect observed in tumor cells (49) and favors aerobic glycolysis over OXPHOS. While this is an inefficient way of generating ATP as compared to the TCA cycle (2 ATPs compared to 36 per glucose molecule), it can be quickly induced which is beneficial when trying to fight microbes that quickly replicate (50). As a result of this metabolic reprogramming, the excess carbon from glycolysis in classically activated macrophages is secreted as lactate instead of being used to produce acetyl-CoA from pyruvate, and the TCA cycle is broken at two points, after citrate, and after succinate, resulting in the accumulation of these two metabolites. The TCA cycle in macrophages has been elegantly reviewed in detail by Ryan and O’Neill (51). A short summary of the most important consequences of metabolic reprogramming is given below.

The first consequence of metabolic reprogramming is a breakpoint of the TCA cycle after citrate, due to lower expression of isocitrate dehydrogenase (**Figure 3B**). This enzyme is responsible for the conversion of isocitrate to α-ketoglutarate and when expressed at lower levels results in more citrate in cells. The accumulated citrate can be transported into the cytosol by mitochondrial citrate carrier family 25 member 1 (SLC25a1) in exchange for malate (52). This carrier is highly expressed in macrophages stimulated by inflammatory signals leading to citrate accumulation in the cytosol (53). Once in the cytosol, citrate can be converted by ATP citrate lyase into acetyl-CoA and oxaloacetate and used for the synthesis of fatty acids, cell membranes and prostaglandins (54), or it can be transported into the nucleus and converted into acetyl-CoA by citrate lyase (55). As the enzyme ATP citrate lyase is upregulated in classically activated macrophages (56) this could lead to higher levels of cellular acetyl-CoA. Acetyl-CoA can then be used for lysine acetylation of proteins, such as histones, by acetyltransferases, therefore having an impact on gene expression (as explained in later paragraphs), or for *de novo* lipogenesis.

The second consequence of metabolic reprogramming is a breakpoint after succinate due to the inhibition of succinate dehydrogenase by competitive inhibitor itaconate, which will result in succinate accumulation (**Figure 3C**). Itaconate is produced by cis-aconitate decarboxylase, also called immune-responsive gene 1, and is present in higher quantities in classically activated macrophages, in which it also induces lactate dehydrogenase, contributing to the buildup of lactate (57). In addition, the above-mentioned accumulation of citrate can contribute to succinate accumulation because citrate can be converted to cis-aconitate in mitochondria and can then be further converted to itaconate by cis-aconitate decarboxylase. The levels of succinate are also increasing as a consequence of increasing levels of glutamine anaplerosis. This means that glutamine is converted *via* α-ketoglutarate into succinate through glutaminolysis or through an upregulated γ-aminobutyric acid (GABA) shunt. This shunt is a TCA cycle bypass which uses glutamine as a substrate to produce succinate, passing through glutamate, GABA, and succinic semialdehyde (58). Incidentally, succinate dehydrogenase is also the second complex of the mitochondrial respiratory chain, which is a series of enzyme complexes that transfer electrons inside the mitochondrial matrix in exchange for protons, that are then pumped out. Succinate dehydrogenase generates ubiquinol from ubiquinone using the electrons obtained from succinate oxidation. Ubiquinol is then reoxidized by complex III, which also reduces cytochrome c, that in its turn will reduce complex IV. This complex then reduces molecular oxygen to water. The transfer of electrons that takes place in the respiratory chain provides the potential energy necessary to generate the proton-motive force required for ATP synthesis (59). Therefore, inhibition of succinate dehydrogenase by itaconate could also lead to decreased mitochondrial respiration in macrophages, providing a link between these two pathways, and leading to higher ROS production due to reverse electron transport to complex one, rather than complex III, that will receive the electrons from ubiquinol and generate NADH from NAD^+^ (60).

Higher levels of succinate also lead to stabilization of transcription factor hypoxia-inducible factor 1-alpha (HIF-1α) because succinate inhibits prolyl hydroxylase domain proteins, which normally hydroxylate HIF-1α leading to its ubiquitination and proteasomal degradation (48). The stabilization of HIF-1α will lead to its binding to hypoxia response elements on target genes and induce their expression. HIF-1α regulates expression of genes associated with angiogenesis, proliferation, inflammation, and cellular metabolism. HIF-1α in fact promotes the switch to glycolysis by inducing expression of glycolytic enzymes like hexokinase 2, glucose-6-phosphate isomerase, and pyruvate kinase M2. These are involved in the first, second, and tenth reactions of glycolysis respectively, to ensure these cells can continue to produce ATP when oxygen is limited. Glycolysis will also supply metabolic intermediates, like glucose-6-phosphate, to the pentose phosphate pathway to produce NADPH (61). This can then be used by the enzyme NADPH oxidase to produce ROS. Members of the oxidative phase of the pentose phosphate pathway (from the entrance of glycolytic glucose-6-phosphate to the production of ribulose-5-phosphate) are all upregulated in classically activated macrophages (62), while members of the nonoxidative phase are downregulated due to downregulation of sedoheptulose kinase, which converts sedoheptulose in sedoheptulose-7-phosphate (63).

HIF-1α also promotes expression of lactate dehydrogenase, which metabolizes pyruvate to lactate (64) and expression of pyruvate dehydrogenase kinase 1. The latter inhibits pyruvate dehydrogenase, therefore inhibiting the conversion of pyruvate in acetyl-CoA, repressing mitochondrial function even more (65).

In summary, highly active glycolysis combined with increased glucose uptake results in improved availability of glycolytic intermediates, meeting one of the requirements of an inflammatory response, such as an increased demand for energy (66).

### Metabolic Reprogramming of Macrophages in DMTII and Obesity

The exact mechanism of how macrophages reprogram their metabolism after activation in DMTII and obesity is still unknown. As discussed in the previous paragraph, macrophages undergo metabolic reprogramming when classically activated, but also availability of energy sources influences their metabolism, implying that abundant availability of energy sources (for instance during hyperglycemia or obesity) can also impact their metabolism. Since it has been shown that the phenotype of metabolically activated macrophages differs from classically activated macrophages (35, 36), this may indicate that their metabolism could differ as well.

**Figure 4:**
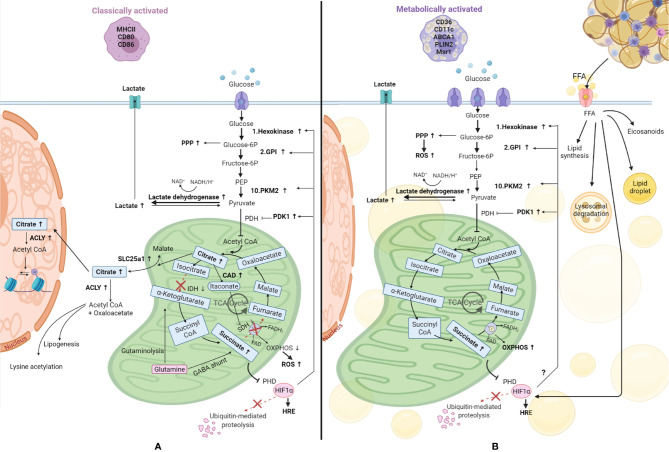
Comparison of metabolic reprogramming of classically and metabolically activated macrophages. **(A)** Classically activated macrophages and their metabolism. **(B)** Metabolically activated macrophages and their metabolism. Adipose tissue macrophages in obesity internalize free fatty acids (FFA) and lipids from the dying adipocytes, becoming foam cells. These FFA can be used to synthesize new lipids, can be stored in lipid droplets, can be catabolized through the lysosomal pathway or be used to produce inflammatory lipid mediators called eicosanoids. Glucose is the main source of energy also in metabolically activated macrophages, where the glucose transporter is overexpressed, and it is catabolized by glycolysis, which is upregulated, providing substrates for the pentose phosphate pathway (PPP), also upregulated. Also the metabolic pathway OXPHOS (oxidative phosphorylation) is upregulated in these cells, underlying their high energy demand. Succinate production is increased in these cells. This metabolite inhibits prolyl hydroxylase domain (PHD) proteins, resulting in less hydroxylation of hypoxia-inducible factor 1-alpha (HIF-1α), which circumvents its degradation, allowing its binding to hypoxia response elements (HRE) on target genes (48). HIF-1α is also promoted by FFA and we hypothesize it might promote the switch to glycolysis by inducing glycolytic enzymes as it happens in classically activated macrophages. TCA, tricarboxylic acid cycle; SLC25a1, solute carrier family 25 member 1; ACLY, ATP citrate lyase; IDH, isocitrate dehydrogenase; SDH, succinate dehydrogenase; CAD, cis-aconitate decarboxylase; GABA, γ-aminobutyric acid; PHD, prolyl hydroxylase domain; ROS, reactive oxygen species; PKM2, pyruvate kinase M2; GPI, glucose-6-phosphate isomerase; PEP, Phosphoenolpyruvate; PDH, Pyruvate dehydrogenase; PDK1, pyruvate dehydrogenase kinase 1; FAO, fatty acid oxidation.

This situation is particularly relevant in DMTII and insulin resistance for adipose tissue macrophages. Similar to classically activated macrophages, metabolically activated macrophages have higher glycolytic rates and produce more lactate (**Figure 4**) compared to adipose tissue macrophages from lean subjects (11). The glucose transporter GLUT1 is overexpressed in metabolically activated adipose tissue macrophages of obese mice and rats (67) and overexpression of this transport was shown to drive more glycolysis and pentose phosphate pathway metabolism in these cells, in addition to higher ROS production (67). Moreover, a higher level of glucose present in the environment can affect macrophage activation state, inducing a phenotypic switch to CD11c^+^ macrophages (68). It has been shown that CD11c+ macrophages have enriched expression of genes encoding glucose metabolism, fatty acid metabolism, and lysosomal proteins (39) emphasizing a central role for these genes in adipose tissue macrophages.

Lipid metabolism also seems to differ between metabolically and classically activated macrophages. As mentioned before, macrophages in crown-like structures in fat tissue internalize the free fatty acids and lipids released from dead adipocytes, thereby becoming foam cells (40). Xu et al. demonstrated that obesity induces lysosomal biogenesis and lipid metabolism pathways in adipose tissue macrophages due to the higher accumulation of lipids in these cells, events that do not take place in classically activated macrophages (35). Adipose tissue macrophages from obese subjects also metabolize lipids differently and a detailed description has been compiled by Dahik et al. (69). A combination of studies has shown that adipose tissue macrophages from obese subjects can synthesize lipids from free fatty acids, store them in lipid droplets, catabolize them through the lysosomal pathway or produce inflammatory lipid mediators called eicosanoids (69, 70). In contrast, adipose tissue macrophages from lean subjects take up free fatty acids and subject them to oxidation (71). The higher levels of free fatty acids, coupled with higher levels of other inflammatory mediators like TNFα, IL6, and IL-1β will lead to the development of insulin resistance which can develop in obesity.

In accordance with their activated status, metabolically activated macrophages were also shown to contain a higher number of mitochondria and have higher mitochondrial activity compared to adipose tissue macrophages from lean subjects (39, 72) and this was associated with higher OXPHOS activity (11, 72). In addition, expression of insulin-like growth factor 1 receptors (IGF1R) was shown to be suppressed in adipose tissue macrophages from obese subjects (73), altering the insulin receptor pathway leading to lower phosphorylation of IRS1-2, lower PI3K activation, and decreased Akt serine phosphorylation. Lower Akt phosphorylation translates into lower mTOR activity and activation of glycolysis (45).

Another major characteristic of adipose tissue in obesity is the hypoxia present and therefore HIF-1α may be overexpressed in adipose tissue macrophages due to oxidative stress, due to a higher content of the metabolite succinate (72, 74), due to the higher levels of free fatty acids (72), or a combination thereof. Therefore, one could speculate that the glycolytic enzymes that are induced by HIF-1α in classically activated macrophages are also induced in metabolic syndrome, but more studies on this topic are needed.

In summary, the metabolic changes found in metabolically activated macrophages resemble the ones that occur when macrophages polarize toward a pro-inflammatory phenotype but rely mostly on high levels of free fatty acids present in the microenvironment and differ in the induction of oxidative phosphorylation.

## Metabolic Changes, Lysine Acetylation and Gene Expression

In recent years it has also become apparent how variations in metabolite levels due to the onset of disease are connected to epigenetic modifications, which lead to changes in gene expression (75).

As mentioned above when discussing the interruptions in the TCA cycle, levels of acetyl-CoA may change due to changes in macrophage metabolism. Acetyl-CoA is generated in the mitochondrial matrix from pyruvate by the pyruvate dehydrogenase complex as part of glycolysis, by β-oxidation of fatty acids, or by the catabolism of branched-chain amino acids. Mitochondrial acetyl-CoA enters the tricarboxylic acid cycle and is converted to citrate (76). It can then be transported out of the mitochondria and reconverted to acetyl-CoA, thus contributing to cytoplasmic protein acetylation. It can also be transported into the nucleus as citrate and reconverted to acetyl-CoA by ATP citrate lyase to serve as a substrate for lysine acetyl transferases.

In addition to being a metabolic intermediate, acetyl-CoA is also a substrate used by lysine acetyl transferases to reversibly transfer an acetyl group to the ϵ-amino group of lysine residues in target proteins. Therefore, altered levels of acetyl-CoA resulting from macrophage metabolic reprogramming will result in different levels of lysine acetylation (77). Acetylation neutralizes the positive charge on lysine, altering the way the acetylated protein interacts with surrounding proteins and other molecules, most notably histones (78). This highlights an interesting and yet still not widely studied consequence of changes in macrophage metabolism: the effect this can have on protein acetylation and consequently on gene expression.

Reversible protein acetylation regulates a number of important cellular processes including gene expression *via* acetylation of histones. In fact, acetylation is one of the most frequent reversible posttranslational modifications histone proteins are subjected to. Other posttranslational modifications include methylation, phosphorylation, and ubiquitylation, which all regulate gene expression by influencing the folding of chromatin. Chromatin is a complex of DNA wrapped around an octamer of histone proteins, one H3/H4 tetramer, and two H2A/H2B dimers, forming nucleosomes (79). During activation of gene transcription, the chromatin conformation changes from tightly packed to relaxed allowing DNA-binding proteins to interact with the DNA. Histone acetylation favors gene transcription because interactions of positively-charged amino groups in histones (belonging to lysine residues) with negatively charged phosphate groups in DNA will decrease due to the removal of positive charges on histones upon acetylation. Interestingly, protein acetylation also regulates the activity of enzymes involved in cellular energy metabolism such as hexokinase, pyruvate kinase isozymes M2, and pyruvate dehydrogenase (77).

### Protein Acetylation in Obesity and DMTII, Immunomodulatory Epigenetics as New Therapies

Due to the changes of intracellular acetyl-CoA concentrations in obesity and DMTII, an effect on protein acetylation and therefore posttranslational protein modifications (i.e. epigenetic modifications) seems probable. Indeed, a link between epigenetic changes and DMTII-related meta- inflammation has been reported in a variety of studies, suggesting that metabolic changes induced by obesity/DMTII can lead to epigenetic changes, which then result in transcription of pro-inflammatory genes (80, 81).

In particular, changes in the levels of lysine deacetylases (KDACs), enzymes responsible for the deacetylation of lysine residues in proteins, have been reported in obese mice and patients (82, 83). KDACs can be classified into four groups (84): Class I comprises KDAC1, 2, 3, and 8; Class II is divided into two sub-groups, IIA (KDAC4, 5, 7, 9) and IIB (KDAC6 and 10); Class III includes the sirtuins, which differ from the other KDACs because they depend on NAD for their deacetylase activity instead of being zinc-dependent like the others; Class IV encompasses KDAC11.

Bricambert et al. have described how lower activity of KDACs, KDAC5 and 6 in particular, in white adipose tissue of obese mice and patients correlated with higher levels of pro-inflammatory adipokines (hormones and cytokines secreted by adipocytes) and with impaired glucose uptake (82). However, other studies have shown that sirtuins are probably more important in regulating metabolism (83, 85). SIRT1 appears to be most closely linked to the metabolic syndrome and is primarily affected by changes in nutrient conditions like caloric restriction (86) or overnutrition. Cao et al. showed that SIRT1 is intimately connected to insulin resistance by regulating insulin signaling and therefore metabolism of glucose and lipids (87). Yeung et al. reported that SIRT1 inhibited inflammatory responses by deacetylating the p65 subunit of transcription factor Nuclear Factor kappa b (NFkB) (88). NFkb regulates a number of processes involved in inflammation, including induction of the expression of pro-inflammatory genes in many cells (89). A negative correlation between SIRT1 gene expression levels and BMI values of patients (90) was previously shown and this was also associated with more pro-inflammatory gene expression contributing to insulin resistance. Similarly, SIRT1 levels were also inversely proportional to infiltration of adipose tissue macrophages in human subcutaneous fat (91). This finding was confirmed *in vitro* by studies showing that SIRT1 inhibits recruitment of macrophages by co-culturing them with SIRT1-deficient adipocytes and showing that the absence of SIRT1 induced their recruitment and a pro-inflammatory phenotype (92). In addition, lower mRNA levels of SIRT1were detected in macrophages of mice fed a high-fat diet that developed obesity (92).

These findings on KDACs suggest that combining KDAC activators with anti-diabetic drugs could be a more efficient way to treat metabolic syndrome. A wide array of KDAC activators is already available and more specific ones are being developed. Examples of KDAC activators that have been used in the context of metabolic syndrome show beneficial effects by inhibiting expression of pro-inflammatory cytokines in adipocytes and higher insulin sensitivity and glucose uptake after treatment (81). Importantly, a SIRT1 activator (SRT2104) has entered clinical trials for treatment of DMTII (93). Encouraging findings in mice on a high-fat diet preceded this clinical trial, showing for example that SIRT1 over-expressing mice had fewer macrophages in adipose tissue (92). Moreover, treatment of RAW264.7 macrophages and primary intraperitoneal macrophages with SIRT1 activators inhibited inflammatory responses to LPS (94). The same treatment in Zucker fatty rats induced a shift from pro- to anti-inflammatory behavior in adipose tissue macrophages, in addition to improved glucose tolerance (94).

In addition to activating KDACs, deacetylase inhibitors have also been used in the context of chronic inflammatory diseases, like chronic obstructive pulmonary disease (95, 96), rheumatoid arthritis (97), and cancer (98) with positive outcomes. This suggests that inhibiting certain deacetylases associated with chronic inflammation in diabetes and obesity may also have therapeutic potential. Inhibitors of Class I KDACs, particularly KDAC3 (99) have been used *in vitro* and *in vivo* in the context of diabetes and obesity (100–102). None of them have reached the stage of clinical trials yet, even though promising results have been achieved in glycemic control and reduction of obesity, highlighting their potential as therapeutic treatment for metabolic disorders.

The above paragraphs have highlighted that macrophages, inflammation and metabolic changes are intimately connected in DMTII and obesity. However how metabolic pathways in macrophages are changed is still unclear and many knowledge gaps remain. To be able to address the lack of knowledge on which metabolic pathways are affected by reprogramming, different techniques must be used and the results integrated. In the following paragraph, the most common ones will be described along with advantages and disadvantages and suggestions which would be most appropriate to use when.

## Analytical Methods to Characterize Macrophage Metabolic Reprogramming

The most widely used method to characterize macrophage polarization and the corresponding phenotypes is flow cytometry (103). This technique determines properties of single cells by assessing the presence of proteins on the surface of cells or intracellularly with fluorescently-labeled antibodies using laser-induced excitation of the fluorescent labels. However, this approach does not provide information on cellular metabolism. Recent work has tried to fill this gap by using flow cytometry to investigate single-cell metabolism using antibodies against key metabolic enzymes (104). Although this work is a major step forwards, it still does not provide quantitative insight into metabolite production and enzyme activity, which is why other analytical methods are needed to gain a better mechanistic understanding of macrophage metabolic reprogramming.

Different techniques are used to measure the metabolic status of cells, including extracellular flux analysis, colorimetric/fluorometric enzyme activity assays, and mass spectrometry (MS) based metabolomics and flux analysis (12). These techniques provide complementary information about the metabolic state of cells. We aim to clarify some of their advantages and disadvantages and provide an overview of when to use which method as well as how to combine them.

### Functional Assays

Widely used assays in the field of immunology are extracellular flux analyzers that give a functional readout of glycolytic or mitochondrial metabolic activity by measuring changes in energy metabolism in culture medium of cells (105). Mitochondrial function and respiratory capacity are assessed by measuring the oxygen consumption rate, which correlates with ATP-linked respiration, maximal and basal respiration, and proton leakage. It is a functional, real-time assay that does not measure the level of individual metabolites, but indirectly measures OXPHOS activity. Glycolytic activity can be assessed by using a glycolytic stress assay that forces the cells to use glycolysis by initial glucose starvation followed by glucose administration and ATP synthase inhibition. The extracellular pH is monitored to calculate the acidification rate. When glucose is converted to pyruvate and subsequently lactate during anaerobic glycolysis, H^+^ ions will be produced, shifting the pH of the medium.

These assays have the advantage of being performed in living cells, but they are indirect indicators of changes in metabolic pathways or mitochondrial function, which play a pivotal role in macrophage activation and function as outlined above (106). However, individual metabolite levels or activity of individual enzymes are not quantified emphasizing the need for complementary analytical approaches to understand how metabolic pathways change during macrophage polarization.

### Enzymatic Assays

Key metabolites to follow with respect to the different macrophage phenotypes are listed in **Table 1** and are part of glycolysis and the TCA cycle. The consumption rate of glucose, as the major source of energy for classically activated macrophages, gives an indication of the overall energy requirement. The conversion of glucose to pyruvate by the action of glycolytic enzymes is an indicator of the role of glycolysis and therefore it is of interest to measure some of its intermediates like glucose-6-phosphate, fructose-6-phosphate or phosphoenolpyruvate. Since classically activated macrophages shift pyruvate conversion from acetyl-CoA production to lactate production, it is critical to quantify lactate. In addition, since the TCA cycle is interrupted at two points, due to a reduced activity of isocitrate and succinate dehydrogenases, leading to accumulation of succinate and citrate, these two metabolites should be measured to assess the contribution of the TCA cycle to the overall conversion of glucose to ATP. Another reason for quantifying citrate is that it can be converted to acetyl-CoA, which serves as substrate for lysine acetyltransferases and is thus linked to protein acetylation and the regulation of gene expression (see paragraph “Lysine acetylation links metabolism and gene expression” for further details). Itaconate is another interesting metabolite to measure, since it plays a role in connecting the two breakpoints of the TCA cycle.

**Table 1:** Metabolite analysis in macrophages metabolic reprogramming.

	Extracellular flux analyzers	Enzymatic essays	Mass spectrometry
**Glycolytic activity**	(55, 105, 107–110)		(55, 111)
**OXPHOS activity**	(55, 105, 108–110, 112)		(55, 111)
**Glucose**		(109, 113, 114)	(55, 111, 113, 114)
**Pyruvate**		(115)	(55, 108)
**Glucose-6-phosphate**			(55, 108)
**Phosphoenolpyruvate**			(55)
**Lactate**		(12, 110, 116)	(55, 108, 112, 117)
**Succinate**		(113, 114)	(55, 108, 111, 117–119)
**Citrate**		(110)	(55, 111, 117–119)
**Acetyl-CoA**			(118)
**Itaconate**			(55, 111, 117, 118)

Rows represent key metabolites or metabolic processes that can be measured in order to differentiate macrophage phenotypes. Columns present examples of references that use the different analytical techniques either alone or in combination.

To quantify lactate, an enzymatic assay can be used for *in vitro* studies (12). This assay is based on generating a luminescent signal that is proportional to the lactate concentration in the cell culture medium. Other enzymatic assays with colorimetric or fluorimetric readouts are available for most, but not for all of the metabolites of interest mentioned above (113, 114). Moreover, these assays do not measure metabolite concentrations directly, but make use of enzymatic reactions that oxidize them, generating a product that reacts with a probe, producing a colorimetric or fluorimetric readout. Techniques that allow quantifying a wider range of metabolites simultaneously are therefore of increasing relevance to gain a more comprehensive view of metabolic changes in cells.

### Mass Spectrometric Assays

The main analytical platform that is used to detect and quantify a wider range of metabolites is MS (120–122). Different kinds of separation methods can be coupled to MS to increase the depth of analysis as well as to cover a wider range of compounds. Gas chromatography (GC) and liquid chromatography (LC) are most widely used (123). LC is better suited to measure polar and charged molecules, whereas GC is preferred when investigating short-chain fatty acids, esters, hydrocarbons, and volatile and thermally stable molecules.

Most metabolite measurements are done at a fixed time point, while it is of interest to rather assess turnover rates, as this will provide a more dynamic picture of the contribution of a given metabolic pathway in, for example, central carbon metabolism. Such measurements can be done using stable-isotope-labeled metabolic substrates (see paragraph “Mass spectrometric metabolic flux analysis”).

Metabolite analyses by MS can be performed in a targeted or untargeted manner. With targeted MS methods it is possible to have accurate and precise quantitative information on known metabolites, such as those that are involved in metabolic reprogramming of macrophages. While many metabolites, that play a key role in macrophage metabolic reprogramming, are known, recent work shows that new metabolites are still being discovered using untargeted MS (124). One example is the discovery of the role of uridine diphosphate N-acetylglucosamine in macrophage polarization (125). Once such a metabolite has been discovered, it may be further investigated in greater detail with a targeted MS approach.

### Mass Spectrometric Metabolic Flux Analysis

Knowing the concentration and/or abundance of the metabolites at a given moment in time can help to understand which metabolite levels are altered at the moment of metabolite extraction. However, to understand the dynamics of metabolic reprogramming, it is also relevant to know which metabolic pathways are up- or down-regulated, which can be deduced from measuring production and consumption rates of metabolites by following the incorporation of stable isotopes into metabolites over time. This is accomplished by using stable isotope labelled metabolic tracer molecules to study the flux of stable isotopes through different pathways for a set of key metabolites (126). An untargeted LC-MS metabolomics flux analysis approach with stable isotope labeling of metabolites was used to get more insight into substrate use in different macrophage phenotypes after stimulation with LPS or IL4 (124). A change in phenotype due to a certain stimulation was first confirmed by flow cytometry and then different metabolic pathways were followed by adding isotopically labelled substrates. This study confirmed that LPS-stimulated macrophages use citrate to synthesize itaconate and transport it to the cytosol to produce lipids. The study established further that IL4-stimulated macrophages rely on oxidative metabolism as their main energy source (see **Figure 3**). Furthermore, the study showed that some metabolites, like itaconate, are only synthesized in mitochondria, while others were produced by both cytosolic and mitochondrial enzymes, depending on the polarization status of macrophages. The integration of different levels of biological information has been used by Jha et al. in a pipeline named “concordant metabolomics integration with transcription” (CoMBI-T) that integrates MS metabolomics data with RNA-seq results in order to characterize macrophage polarization (125). Polarization was first confirmed by flow cytometry and then metabolic profiles were acquired using non-targeted MS-analysis. Altered metabolic pathways were further studied by metabolite flux analysis using ^13^C labeled glucose and ^13^C- and ^15^N-labeled glutamine combined with a targeted LC-MS approach (selective reaction monitoring (SRM)). Using this pipeline they showed that glutamine is a major source of nitrogen in alternatively activated macrophages and that these cells have an augmented metabolism of amino sugars and nucleotide sugars like uridine diphosphate N-acetylglucosamine. This metabolite is known to link signaling and metabolism through glycosylation of proteins that are localized at the cell-surface, for example, various growth factor receptors (127). By using this combined approach a new metabolite was found, which plays a major role in alternatively activated macrophages (125). The authors also investigated breakpoints in the TCA cycle using metabolic flux analysis experiments and found that the aspartate-argininosuccinate shunt, a series of reactions that connect the TCA cycle and the urea cycle (128), plays a role in pro-inflammatory macrophages.

To summarize, a number of analytical approaches can be used to study metabolic reprogramming in macrophages. Flow cytometry analysis allows to define the different phenotypes of macrophages on which subsequent comparative analyses are based. Extracellular flux analysis provides functional insight into macrophage metabolic reprogramming at the level of metabolic pathways that are altered but does not quantify individual metabolites. Enzymatic assays with luminescent, colorimetric or fluorimetric readouts quantify a number of key metabolites. These assays are relatively easy to perform and data analysis does not require specific expertise as is the case for MS analysis. MS in combination with GC or LC allows to cover a wider range of metabolites both known and unknown. It thus allows to follow known key metabolites as well as to potentially gain insights into new metabolites or metabolite patterns. MS analysis can be extended to comprise metabolic flux analysis in order to follow how metabolites are consumed and produced in cell systems and how this varies in the context of changing conditions, for example during the development of DMTII. It is thus of particular interest to combine multiple analytical approaches to gain a comprehensive overview of mechanisms related to macrophage metabolic reprograming. Moreover, even though there is considerable knowledge about metabolic reprogramming during macrophage polarization in cell culture under defined conditions, there is still a large knowledge gap when it comes to diseases like DMTII. Here metabolic changes are key disease features, which most probably will also reflect in changes in the metabolism of macrophages and their polarization. That is why metabolomics in combination with lipidomics, fluxomics, transcriptomics, and proteomics has the potential to lead to the discovery of further mechanistic links between inflammation and metabolic disturbances (129).

## Future Perspectives

**Figure 5:**
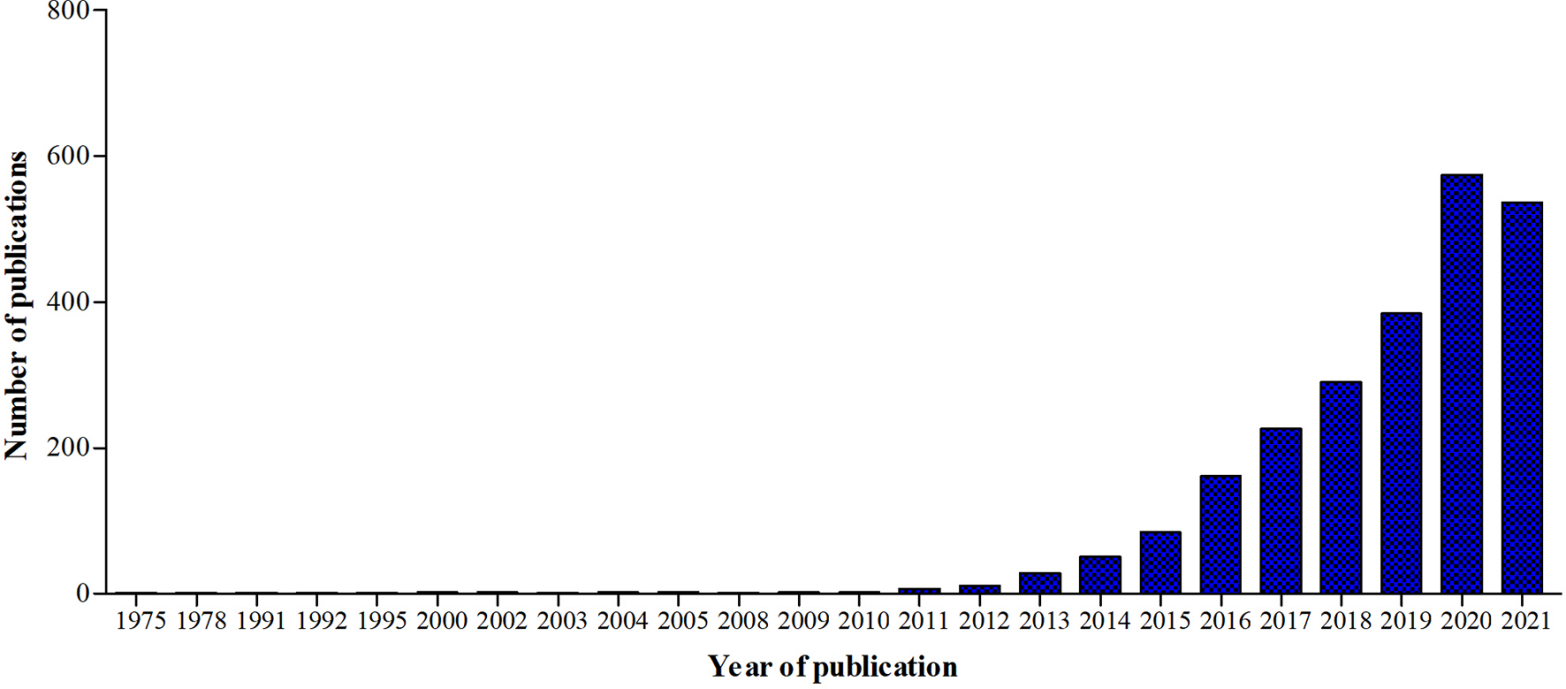
Graphic representation of the number of publications in Pubmed.gov using the keyword ‘Immunometabolism’ from 1975 until 2021.

A new branch of immunology, called immunometabolism, has been developing rapidly in the past ten years (**Figure 5**). It is defined as the interplay between immunological and metabolic processes and has solid foundations in macrophage biology research, which is illustrated by the fact that 29% of the 2027 publications since 1975 also had the key word ‘macrophages’.

We now have clear evidence that macrophages can change metabolic pathways to respond to challenges they encounter. Macrophage polarization and function are highly dependent on fast changes in intracellular metabolism, which explains why macrophages can be extremely versatile in function. While this field is moving forward fast, our understanding of interactions between metabolic changes at the organism level such as found in DMTII/obesity and intracellular metabolic changes in macrophages still lags behind. Macrophages in adipose tissue from individuals with DMTII or obesity clearly show a different but pro-inflammatory phenotype from those of lean individuals or individuals without DMTII. However, those pro-inflammatory phenotypes appear to be unique to DMTII and obesity and are not found when macrophages are M1-polarized in acute inflammation (36), suggesting that metabolic changes at the organism level influence macrophage activation and metabolism. How they interact exactly remains an open question that could potentially be answered by improved analysis of macrophage cellular metabolism. To do this in complex conditions like DMTII and obesity, an approach from different angles is needed and integration of results from different analytical platforms is the way forward. This could help to better understand the mechanism underlying macrophages polarization in DMTII-related chronic inflammation and, even more importantly, how this affects disease progression.

A better understanding of interaction between different metabolic pathways could also result in the development of new treatment options. Diabetes treatment now focuses on weight loss, rebalancing insulin resistance, and lowering blood glucose levels by using mostly gluconeogenesis inhibitors like metformin or hypoglycemic drugs that stimulate β-cells to release insulin (130). While weight loss should always remain the number one priority, redirecting adipose tissue macrophage metabolic programs, and thereby polarization, could be another interesting approach since pro-inflammatory macrophages contribute to onset of insulin resistance (131) and overall to DMTII-related meta-inflammation. Inhibiting chronic inflammation, would not only improve the comorbidities, like diabetic retinopathy, polyneuropathy, or nephropathy but also one of the main causes of diabetes onset.

Another aspect that should be further investigated and could develop into a promising therapeutic approach is the link between metabolism and inflammation *via* epigenetic regulation of gene expression. Recent studies indicate that chronic inflammation is linked to changes in energy metabolism *via* lysine acetylation of both histones and non-histone proteins (75). Therefore, deacetylase inhibitors or activators may be additional approaches to inhibit macrophage-induced meta-inflammation by rebalancing the expression of pro- and anti-inflammatory mediators.

## Author Contributions

SR, MK, NG, RB, and BM conceived and designed the set-up of the manuscript. SR drafted the manuscript and prepared figures. SR, MK, NG, RB, and BM edited and revised the manuscript and all approved the final version. All authors contributed to the article and approved the submitted version.

## Funding

This project has received funding from the European Union’s Horizon 2020 research and innovation program under the Marie Skłodowska-Curie grant agreement No 754425.

## Conflict of Interest

The authors declare that the research was conducted in the absence of any commercial or financial relationships that could be construed as a potential conflict of interest.

## Publisher’s Note

All claims expressed in this article are solely those of the authors and do not necessarily represent those of their affiliated organizations, or those of the publisher, the editors and the reviewers. Any product that may be evaluated in this article, or claim that may be made by its manufacturer, is not guaranteed or endorsed by the publisher.
